# Trends in the Genetic Background of Methicillin-Resistant *Staphylococcus Aureus* Clinical Isolates in a South African Hospital: An Institutional-Based Observational Study

**DOI:** 10.2174/1874285801711010339

**Published:** 2017-11-30

**Authors:** John F. Antiabong, Marleen M. Kock, Tsidiso G. Maphanga, Adeola M. Salawu, Nontombi M. Mbelle, Marthie M. Ehlers

**Affiliations:** Department of Medical Microbiology, University of Pretoria, Gauteng, South Africa

**Keywords:** MRSA, Diversity, Epidemiological genetics, Hospital setting, South Africa

## Abstract

**Background:**

This study sought to understand the epidemio-ecological dynamics of MRSA isolates associated with a South African hospital over a period spanning year 2007-8 (a previous study reported in 2009) and year 2010-11 (this study).

**Methods:**

One hundred and ninety three isolates were characterised by molecular fingerprinting methods including pulsed field gel electrophoresis (PFGE), *spa* typing, *agr*-typing, SCC*mec*-typing, and multilocus sequence typing (MLST). The Vitek-2 automated antibiogram of representative isolates was also performed.

**Results:**

Our data shows that the distribution of MRSA strains among the different clinical conditions was rarely dependent on the genetic backbone or genotype. Compared to the previous survey in 2009, CA-MRSA isolates increased by 31% while HA-MRSA isolates decreased by 17%. An increase in genetic diversity was also revealed including the detection of three pandemic clonal complexes (*spa* type t012-ST36/CC30, *spa* type t037-ST239/CC8, *spa* type t891-ST22/CC22 and *spa* type t1257-ST612/CC8). Majority of the genotypes were classified as Spa Cluster B-SCC*mec* I-*agr* I 19.2%; (37/193) Spa Cluster A-SCC*mercury*-*agr* I 14.5%; (28/193)

**Conclusion:**

This study reveals that increased diversity in MRSA genetic background was associated with resistance to frontline antibiotics. Also, an increase was recorded in the CA-MRSA/HA-MRSA ratio within a 5-year period despite the continuous dominance of the HA-MRSA genotype.

## 1 INTRODUCTION

*Staphylococcus aureus* is accountable for a high proportion of cases of severe infection in hospital and outpatient units [1]. Resistance to methicillin and other β-lactam antibiotics, such as penicillin and cephalosporins is caused by the acquisition of the *mec*A gene, which codes for a 78-kDa penicillin binding protein [2]. The epidemiology of MRSA after its discovery has evolved from the initial healthcare-associated MRSA (HA-MRSA) genotypes which were mostly associated with healthcare facilities to include Community- associated MRSA (CA-MRSA) which are associated with community settings [2]. Although cases of HA-MRSA replacement by CA-MRSA in hospital infection have been reported [3], an evolutionary model has suggested that high heterogeneity of MRSA in human populations may drive the eventual co-existence of CA-MRSA and HA-MRSA genotypes [4].

In addition, there have been reports of increased diversity of MRSA genetic background [5, 6] which may have contributed to the blurring of the distinction between CA-MRSA and HA-MRSA [2]. Pathogen evolution modelling has shown that variability in the nucleotide sequence depends on the duration of infection and that a 10% increase in the duration of infection can result in a 10 fold increase in the chance of clonal replacement, which is a risk factor for increased infectivity or a disease outbreak [7].

Infections caused by MRSA are usually associated with high morbidity and mortality therefore, understanding the molecular epidemiology and evolution of MRSA will provide useful information for controlling transmission in healthcare and community settings [8]. To this end, a follow-up investigation of the survey by Makgotlho and colleagues [1] at the same tertiary hospital was conducted and revealed a tendency towards extensive MRSA diversity over time. In the African continent, there is a dearth of investigation on the follow-up of epidemiological genetics as presented in this report. This report describes an in-depth phenotypic, genetic and epidemiological relationship among clinical *S. aureus* isolates in a hospital and suggests that continuous survey of the genetic diversity of *S. aureus* is an important measure in the management and control of the associated infections in the hospital setting.

## 2 MATERIALS AND METHODS

Written consents were obtained from participants, parent or guidance of minors, accordingly the ethics guidelines of the Faculty of Health Science, University of Pretoria and the ethics approval for this study was obtained from the Research Ethics Committee of the Faculty of Health Sciences, University of Pretoria (protocol number S189/2010).

### 2.1 Sample Source

One hundred and ninety three MRSA isolates collected from female and male patients (age: 1 day to 78 y with an average of 39 y) during the period of April 2010 to August 2011, were obtained from the Department of Medical Microbiology, Tshwane Academic Division, National Health Laboratory Service. The *S. aureus* isolates were received as MRSA positive bacteria after routine diagnosis using the Vitek2 system (bioMA(c)rieux, Mary l'Etoile, France). Briefly, the isolates were recovered from the clinical specimen using the chromogenic agar (MRSA select) (Bio-Rad Laboratories, USA), before identification with the Vitek2 system. Bacterial genomic DNA extraction from the 193 MRSA isolates was performed using the ZR Fungal/Bacterial DNA MiniPrep (Zymo Research, Thermo Scientific, USA), according to the manufacturer's instruction that was modified by the addition of Iβ-mercaptoethanol (reducing agent) to the Fungal/Bacterial DNA binding buffer to a final concentration of 0.5% (v/v).

A multiplex PCR (M-PCR) assay targeting the 16S rRNA (*S. aureus* genus specific detection), *mec*A and *luk*-PV genes was performed using specific primer sets Table (**1**) as previously described [9]. Genomic DNA from a CA-MRSA strain (ATCC CA05) served as a positive control. The M-PCR amplicons were fractionated at 100 V/cm in a 1% (m/v) MetaPhor^TM^ agarose gel (Lonza, Rockland, USA) containing 5 Aµl of ethidium bromide (10 mg/ml) (Promega, Madison, USA) and visualized using an Ultra Violet light box (DigiDoc, UVP product, Upland, California).

The SCC*mec*-typing and CA-MRSA and HA-MRSA designation were performed Using Specific Primer pairs Table (**1**) as previously described [10] including a PCR internal control (targeting *mec*A) to assess the success of the assay. The PCR amplicons were visualised as described for the 16S rRNA amplicons.

The genetic relatedness of the MRSA isolates was determined using pulsed field gel electrophoresis (PFGE) [11] and the PFGE banding patterns that were analysed [12] The MRSA isolates were s*pa*-typed [13] using specific primers Table (**1**) that targeted the repetitive sequence region (Fc binding region and the X region) of the *S. aureus* protein A gene. Nine representative isolates (2, 71, 100, 131, 133, 134, 143, 165 and 183) were randomly selected from each UPGMA cluster for the *spa-*sequence type determination using specific primers Table (**1**) that targeted X region as previously described [14]. The amplicons sizes were verified in a 0.5 µg/ml ethidium bromide-stained agarose gel electrophoresis (1% agarose in TBE buffer; 60 V; 1 h) using a 100 bp molecular weight marker (Fermentas Life Sciences, Thermo Scientific, USA) and sequenced commercially (Inqaba biotechnical Industries, South Africa).

Multilocus sequence typing (MLST) analysis of representative MRSA isolates from the nine *spa*-typed MRSA isolates targeting seven housekeeping genes Table (**1**) was PCR-amplified using specific primers [15]. The amplicons sizes were verified by agarose gel electrophoresis; purified using the Zymoclean gel DNA recovery kit (Fermentas, Thermo Scientific, USA), and sequenced commercially. The sequences obtained for each of the housekeeping genes were analysed using the CLC main workbench version 6.0 (CLC, Denmark) and uploaded on the MLST database (http://saureus.mlst.net/sql/multiplelocus) where allelic profiles and sequence types were assigned.

The PCR assay for *agr-*typing consisted of a forward primer (used for all reactions) and four reverse primers (*agr* group I, II, III and IV) Table (**1**) as previously described [16]. The PCR assays were performed in two separate combinations: (i) the forward primer and two reverse primers for *agr* group I and II, and (ii) the forward primer and two reverse primers for *agr* group III and group IV. The PCR amplicons were visualised as described for the 16S rRNA amplicons.

### 2.2 Statistical Analysis

The prevalence of each variable tested was determined using percentages and the MRSA clonal diversity (pulsotypes and *spa* clustering) was determined as previously described [1]. A 70% similarity coefficient was used as a cut-off point for cluster definition with a tolerance of 1.1% and band optimisation of 1.41% for *spa*-clustering analysis. The *spa* types were determined [16] using the Ridom Staph Type software (Ridom GmbH, WA1/4rzburg, Germany). *Z*-test independent proportion groups (https://www.mccallum-layton.co.uk/tools/statistic-calculators) was used to determine the level of significant difference of parametric variables.

## 3 RESULTS

### 3.1 General Observations

Table (**2**) shows the sources and recovery of MRSA isolates investigated in this study. Blood cultures yielded the most isolates [39% (75/193)] followed by pus swabs [23% (44/193)] and central venous pressure (CVP) tips [13% (25/193)]. The contribution of MRSA isolates from other specimen types ranged from 0.5-8%. All the MRSA isolates [100% (193/193)] were positive for the 16S rRNA (756 bp) and *mec*A (310 bp) genes. One [0.5% (1/193)] CA-MRSA isolate was positive for the PVL (433 bp) gene (Fig. **S1A**).

Table (**S1**) shows a comprehensive data obtained in this investigation. Assessment of the data for possible associations between the variables evaluated showed that HA-MRSA was significantly (*p*<0.05) more prevalent in adult female [71% (60/84)] than in adult male [31% (32/103)] patients Table. (**S1**). On the contrary, in paediatric patients (ages 1d to 8y), HA-MRSA was more prevalent in male [72% (23/32)] than in female paediatrics [(26% (16/60)]. Similarly, the CA-MRSA isolates were more prevalent in the adult female [46% (39/84)] than in the adult male patients [28% (29/103)]; however, not statistically significant (*p*>0.05). Moreover, there was no significant difference in the number of CA-MRSA isolates [7% (2/29)] in the male paediatric patients compared to that in the female paediatrics [13% (5/39)].

It was also oberved that 2.1% (4/193) of the isolates were found to be of SCC*mec* type II + IV occuring only in the CA-MRSA genotype, -of *agr* III type, -of pulsotype J isolated from blood cultures and in female patients alone (Table **S1**).

### 3.2 PFGE Analysis

The PFGE fingerprints of two (1%) of the isolates were un-typeable with the *Sma*I restriction enzyme. The clustering of the remaining 191 typeable MRSA isolates revealed eleven clonal patterns Tables (**S1**, **2**, Fig. **S1B**) and 58% (110/191) of the clinical MRSA isolates showed similar finger-prints and were designated pulsotype-A (which included subtypes A1 to A6). The remaining isolates were designated pulsotypes B to K which differed from pulsotype-A by more than seven bands (Fig. **S1B**, Table **2**).

### 3.3 SCC*mec* typing and isolates designation as CA-MRSA or HA-MRSA

Twenty-two per cent (43/193) of the MRSA isolates were un-typeable by the SCC*mec*-typing method, but were positive for the *mec*A [(internal control) gene (147 bp)]. Five per cent (9/193) of the MRSA isolates showed double bands for SCC*mec* type II (398 bp) and SCC*mec* subtype IVc (200 bp). The remaining 73% (142/193) typeable MRSA isolates were characterised as HA-MRSA [65% (92/142)] and CA-MRSA [35.2% (50/142)] Tables (**2**, **S1**). None of the MRSA isolates were positive for SCC*mec* subtype IVc (200 bp) or SCC*mec* type V (325 bp).

### 3.4 *Agr* Typing

A total of 193 MRSA isolates were typeable by the *agr-*typing assay. The isolates were grouped into *agr* I to III including a hybrid (*agr* group I and III) Tables (**2**, **S1**). No *agr* group IV isolates were detected.

### 3.5 *Spa* and *MLST* Typing

Out of the 193 PFGE typeable isolates, 3% (6/193) were un-typeable by *spa-*typing and were excluded in the final *spa*-clustering analysis. Twenty distinct *spa*-clusters designated A to T and seven outliers Fig. (**S2**) were recovered. Majority of the isolates belonged to cluster D [25.6% (46/187)] and A [20.9% (39/187)]. Four *spa* types and *spa*-sequence types were identified and included those with worldwide distribution while isolates 134 and 165 were un-typeable by the *spa-*typing method Table (**2**). The allele number for the *yqi*L gene of isolate 2 was not found on the MLST website as well as the allelic numbers for *arc*C, *aro*E and *pta* gene in isolates 134 and 165 Table (**3**). The Vitek2 system antibiogram data of the *spa-* sequenced/MLST-typed representative MRSA isolates (*n* =10) is presented in Table (**4**).

### 3.6 Antibiotic Susceptibility Test

The Vitek2 automated antibiogram (bioMA(c)rieux, Mary l'Etoile, France) of the representative MRSA isolates that were selected from distinct PFGE clusters revealed that the CA-MRSA isolates were resistant to frontline antibiotics including glycopeptides (vancomycin (MIC ≥ 32), and teicoplanin (MIC ≥ 32),), ciprofloxacin (MIC ≥ 8), rifampicin (MIC ≥ 32), and fusidic acid (MIC % 32) Table (**4**).

### 3.7 Overall Genotypic Distribution and Epidemiology of the Clinical *S. aureus* Isolates Within the Study Site

A number of genetic distribution events were observed among the isolates within the clinical setting of this study Table (**S1**). Summarily, the distribution of MRSA strains among the different clinical conditions was rarely dependent on the genetic backbone or genotype. The genotypes with the highest frequency of occurrence were Spa Cluster B-SCCmec I-agr I [19.2%; (37/193)]; Spa Cluster A-*SCCmercury*-*agr* I [14.5%; (28/193)] and Spa Cluster E-SCC*mercury*-*agr* I [7.8%; (15/193)] (Table **5**).

### 3.8 High level of Variation in the Genetic Background of Clinical *S. Aureus* Isolates from one Hospital

Although 193 (100%) isolates were positive for the *mec*A and *Staphylococcus* genus specific 16S rRNA genes, there were variations in the number of isolates that could be typed using other molecular algorithms including PFGE [99% (191/193)], SCC*mec* [74% (142/193)], *spa* [31% (6/193)], *agr* [100% (193/193)] and MLST of representative isolates from PFGE clustering [100% (9/9)] algorithms. Despite these variations, majority of the isolates clustered into one major pulsotype (58%), as shown by PFGE typing Table (**2**).

## 4 DISCUSSION

The findings in this study are presented in three parts. Firstly, the comparison between the observations in the current study and that reported by Makgotlho and colleagues [1] in the same hospital. The second part discuss the observations that are specific to the current study, while the summary and limitations of the findings are presented in the third part.

### 4.1 Comparison of the Epidemiological Survey of 2007-8 and 2010-11

An in-depth characterization of clinical MRSA isolates revealed significant flux in the epidemiological genetics and dynamics of these pathogens over time when the current data (spanning year 2010-11) was compared to that in the report of Makgotlho and colleague [1], (data spanning year 2007-8). Molecular and phenotypic characterisation of the MRSA isolates showed evidence of increased CA-MRSA; consistently low PVL carriage among MRSA isolates; high HA-MRSA prevalence in females, and CA-MRSA clones resistant to frontline antibiotics. The recovery of MRSA isolates from blood, pus and CVP tips suggest the ability of *S. aureus* to remain viable in diverse environment within and outside the host.

Significant changes in the genetic background of *S. aureus* isolates between the 2009 study (1) and the current study included:

#### i Decrease in the Number of SCCmec II of the HA-MRSA Genotype

The SCC*mec*-typing in this investigation showed that 42% (60/142) [excluding the SCC*mec*-untypeable isolates] of the SCC*mec* II were HA-MRSA whereas in the 2007-8 survey [1], 67% (65/97) of SCC*mec* II were HA-MRSA strains representing a 25% decrease in the SCC*mec*II HA-MRSA genotype. The SCC*mec* II element has been described to include junkyard regions in which resistance determinants for non-Iβ lactams antibiotics are inserted resulting in multidrug resistance phenotypes [3]. The SCC*mec* III genotype is thought to be a composite element consisting of SCC*mec* III and SCC*mercury.* However, 14% of HA-MRSA that were detected in the Makgotlho and colleagues survey [1] was of SCC*mec* III genotype while in this study no SCC*mec* III was detected.

#### ii Emergence of the SCCmercury (Antiseptic Resistance) Genotype

SCC*mercury* genotype appeared to have emerged [22% (31/142)] in place of SCC*mec* III in this study and were all identified as HA-MRSA. It is not certain if this is in response to the increased use of antiseptic cleaning agents in the hospital. Evidence of genetic mixing whereby 5% (9/193) of the isolates showed double bands 398 bp (SCC*mec* II) and 200 bp (SCC*mec* IVc) were observed suggesting a genetic cross between CA-MRSA and HA-MRSA. Similarly, Shittu and colleagues [17] reported HA-MRSA and CA-MRSA isolates that shared the same PFGE pulsotypes. There is an on-going effort in our laboratory to sequence the whole genome of some these isolates for confirmation.

#### iii Increased Diversity of the Encoded Spa Gene in the S. Aureus Isolates

The increase (*p*=0.05) from three *spa-*clusters in the 2009 study [1] to 20 *spa*-clusters (with fewer number of isolates in most clusters) in the current study Fig. (**S2**) suggests: (i) an increase in the *spa* diversity, (ii) a divergent evolutionary trend. These changes indicate an increased diversity of the genetic backgrounds of MRSA recovered from patients attending the hospital from 2007-2011.

#### iv Increase in The Number of SCCmec Un-Typeable Isolates

Using the Zhang and colleagues SCC*mec*-typing method [10], 73% (142/193) of the MRSA isolates were typeable. However, 22% (43/193) of the MRSA isolates were un-typeable using this method. Five percent (9/193) of the isolates showed double electrophoretic bands of the PCR products. The un-typeable MRSA (22%) was higher than that [8% (8/97)] reported by Makgotlho and colleagues [1] (*p* < 0.05). This suggests an increase in MRSA diversity over the period of survey in this report.

#### v Clinical S. Aureus Isolates with Allelic Number Not Found on the MLST Database

Isolates with novel allelic numbers were also identified. This included isolate 2 (allelic number for the *yqi*L gene not found in the MLST database) as well as isolates 134 and 165 (allelic numbers for *arc*C, *aro*E and *pta* were also not found) Table (**3**). Moreover, the allelic numbers for the other MLST-housekeeping genes of isolate 2 were similar to that of clone ST612 [18], which are 3-3-1-1-4-88-83 Table (**3**). Interestingly, isolates 100 (SCC*mec* IVd) and 143 (SCC*mec* IVa) were MLST-classified has ST612/CC8 (3-3-3-34-88-83) and shared the same *spa* type (t1257) with isolate 2. Clone ST612-MRSA-IV has been described as an infrequently encountered clone that has only been reported in Australia and South Africa [19, 20].

#### vi Increased HA-MRSA/CA-MRSA Ratio and Consistently Low PVL Prevalence Within the Period of Three Years

Healthcare-associated MRSA isolates represented 48% (92/193) of all the isolates, with the majority of isolates classified as SCC*mec* type II [31% (60/193)]. Community-associated MRSA isolates represented 35% (68/193) of all the isolates, of which 33% (64/193) were SCC*mec* subtype IVd. All these data are lower than those reported by Makgotlho and colleagues [1] using the same method. The 2009 survey [1] recovered 81% (79/97) HA-MRSA isolates of which 67% (65/97) were SCC*mec* type II and 14% (14/97) of SCC*mec* type III with the exception of CA-MRSA (SCC*mec* subtype IVd) isolates which were lower [4% (4/97)] than in the current study. Overall, over a period of 5 years in the investigated clinical setting, CA-MRSA isolates increased by 31% while HA-MRSA isolates decreased by 17%. This indicates a reduction in the HA-MRSA/CA-MRSA ratio, from 5:1 in the 2007-8 survey to ratio 2:1 in the current study (*p* = 0.05).

It has been suggested that CA-MRSA has a narrow antibiotic resistance spectrum and a lower cost of treatment in the hospital setting compared to HA-MRSA [3]. However, this does not explain the increased number of CA-MRSA observed in the current study suggesting an alternate mechanism for the observed persistence, such as the acquisition of new antibiotic resistance determinants which broadens the antibiotic resistance spectrum [2].

Despite the detection of 4% (4/97) PVL in the 2007-8 survey [1] within in the same healthcare facility, it was interesting to observe a continuous low number of MRSA isolates with a PCR-detectable PVL gene [0.5% (1/193)] in this hospital. This is in contrast with the reported high prevalence of PVL in a Nepalelese hospital (26.1%) and 14.3% in a Chinese hospital [21, 22].

### 4.2 Specific Observations Made in the Current Study

#### i CA-MRSA strains display resistance to frontline antibiotics, habour PVL and of a metabolically fit lineage

All the MRSA that were resistant to frontline antibiotics were of the CA-MRSA genotype. However, a confirmatory E-test on isolates 2 and 71 indicated that these two isolates were sensitive to teicoplanin (0.75 µg/ml) and vancomycin (0.1 µg/ml) and a repeat test not performed. Anecdotal evidence from researchers indicate that E-test and Vitek results for teicoplanin and vancomycin do not always correlate indicating a need to revisit the use of Vitek automated system for these antibiotics.

Interestingly, the PVL positive CA-MRSA in this study was of the ST22/CC22 lineage and further characterised as PFGE A3-SCC*mec*IV-*spa* type t891/ClusterD-*agr*I. Knight and colleagues [22] showed that the ST22/CC22 lineage is fitter; possesses the ability to acquire antibiotics resistance determinants; replaces other established MRSA clonesas well as being a dominant (CC22 SCC*mec* IV clone) disease-causing MRSA.

#### ii Alternate HA-MRSA Gender Predominance in Adult vs 1-8 Year Old Paediatric Patients

This study revealed potential gender predominance for both genotypes of MRSA. It was found that CA-MRSA and HA-MRSA were more prevalent in adult female than male and *vice versa* in paediatric patients. These findings corroborate with an Ethiopian survey [female (85.2%); male (14.8%)] [23]. It must however be noted that this study did not investigate factors that may have driven the observed gender predominance which could be multi-factorial and beyond the primary focus of this report thereby, warranting an in-depth investigation.

#### iii The Presence of Clonal Complexes and Sequence Types Previously Associated with Pandemics, Resistance to Multiple Antibiotics and Successful Epidemiological Spread

The current data highlighted a number of potential genetic and epidemiologic arsenals of MRSA success in the assessed healthcare setting:

Isolates 131 and 133 were characterized as PFGE A-SCC*mec* (II+SCC*mercury*)-*spa* type t037/ClusterB-*agr* III and PFGE A-SCC*mec* IVa-*spa* type t037/ClusterB-*agr*I respectively and assigned to the ST239/CC8) clone which has been associated with pandemic outbreaks; resistant to multiple antibiotics, and accounts for 90% of all HA-MRSA in Asia and South America [4, 13]. Moreover, the pandemic clone t037-ST239-MRSA-III has previously been reported in Cape Town and Pretoria cities; KwaZulu-Natal province of South Africa and Austria [18, 24, 25, 29] and ST239-IV reported in Iran [29] It is worthy of note that the sequence type 239 is thought to have evolved from clone ST8 [13] and able to withstand background mutations, a trait that ensures its success in coping with different nosocomial ecosystems [26].

More importantly, the detection of MRSA sequence types corresponding to the three pandemic clonal complexes (spa type t037-ST239/CC8, spa type t1257-ST612/CC8, spa type t891-ST22/CC22 and spa type t012-ST36/CC30) is indicative of a potential risk factor for an outbreak of MRSA infection as these clonal complexes have been reported worldwide [4, 19, 23]. In addition, the detection of an epidemic clone [t891-ST22/CC22 (EMRSA-15)] harbouring the PVL gene (which enhances the virulence of this already highly transmissible clone) in the clinical setting is alarming as large nosocomial outbreaks of PVL positive ST22/CC22 have been reported in Germany, Hong Kong and India [13]. Therefore, in this report, the detection of MRSA clones (ST22/CC22 and ST36/CC30) with the reported ability to cause nosocomial transmission and infection [19] is disturbing. Isolate 183, was identified by MLST typing as ST36/CC30 and genotyped as PFGE J-SCC*mec*II-*spa* type t012/Cluster I-*agr*I and III. The ST36 is also referred to as EMRSA-16 (USA200) a robust clone known to cause infections in Australia, Belgium, and South Africa [27].

#### iv Genotypic Distibution Events Within the Study Site

The MRSA genetic distribution events described in the result section above Table (**S1**) indicates a high genetic diversity of MRSA strains across several units of the hospital. Moreover, the presence of specific MRSA strains in a hospital unit was rarely dependent on the genetic backbone or genotype Table (**S1**). It will be interesting to investigate whether these observations indicate a Darwinian evolutionary mechanism for biological fitness in terms of virulence and/or increase in antimicrobial resistance spectrum.

### 4.3 Summary and Study Limitations

Community-associated MRSA is reported to be more virulent, spread rapidly and able to cause different clinical syndromes [2]. Therefore, whether the observed increase in CA-MRSA/HA-MRSA ratio is suggestive of a gradual replacement of the HA-MRSA genotype in *S. aureus*-associated disease or, a means towards the establishment of a balance in the population of both genotypes in order to ensure persistence in the human host, is yet to be unravelled.

Although only the *spa*-typed MRSA isolates were randomly selected for the MLST typing, three pandemic clonal complexes could be identified among the MRSA isolates. While this is epidemiologically relevant, the authors acknowledge that the derived MLST classification may not necessarily be representative of all other isolates in the same *Spa* cluster. The PFGE was anlaysed using the method described by Tenova and colleagues [12], while this method was able to differentiate the clinical *S. aureus* isolates based on their genetic content, it did not allow the assessment of the genetic relationship between the genotypes. A limitation in this study is the lack of in-depth clinical data of the patients from which the samples were obtained. This would have allowed for correlation between the molecular and clinical data. In addition, the definition of CA-MRSA and HA-MRSA was based on molecular principles alone [28] and did not include the clinical criteria. Despite the small sample size, this report forms the basis for similar studies in other African nations.

### CONCLUSION

In conclusion, this study reveals an increase in the CA-MRSA/HA-MRSA ratio (within a 5 year period) despite the continuous dominance of the HA-MRSA genotype. More importantly, the increased diversity in MRSA genetic background was associated with resistance to frontline antibiotics. A similar trend regarding the increase in CA-MRSA genotype and MRSA diversity has been previously reviewed however, with no such data from the African continent [2]. An in-depth phenotypic, genetic and epidemiological correlation as described in this report is a necessary step towards understanding the diversity and epidemio-ecological dynamics of clinical *S. aureus* isolates in African hospitals.

## Figures and Tables

**Table 1:** List of primers for the molecular characterization of MRSA isolates and percentage of MRSA-associated samples recorded.

**MRSA identification**				
**Primer**	**Oligonucleotide Sequence (5'- 3')**	**Target gene/position in genome**	**Amplicon Size (bp)**	**Reference**
Staph 756F Staph 756R	-AACTCTGTTATTAGGGAAGAACA- -CCACCTTCCTCCGGTTTGTCACC-	16S rRNA	756	McClure *et al*., 2006
MecA1-F MecA2-R	-GTAGAAATGACTGAACGTCCGATAA- -CCAATTCCACATTGTTTCGGTCTAA-	*mec*A	310	
Luk-PV-1F Luk-PV-2R	-ATCATTAGGTAAAATGTCTGGACATGATCCA- - GCATCAAGTGTATTGGATAGCAAAAGC-	*lukS/*F-PV	433	
**SCC*mec* typing**				
Type I-F Type I-R	-GCTTTAAAGAGTGTCGTTACAGG- -GTTCTCTCATAGTATGACGTCC-	SCC*mec* I	613	Zhang *et al*., 2005
Type II-F Type II-R	-CGTTGAAGATGATGAAGCG- -CGAAATCAATGGTTAATGGACC-	SCC*mec* II	398	
Type III-F Type III-R	-CCATATTGTGTACGATGCG- -CCTTAGTTGTCGTAACAGATCG-	SCC*mec* III	280	
Type IVa-F Type IVa-R	-GCCTTATTCGAAGAAACCG- -CTACTCTTCTGAAAAGCGTCG-	SCC*mec*IVa	776	
Type IVb-F Type IVb-R	-TCTGGAATTACTTCAGCTGC- -AAACAATATTGCTCTCCCTC-	SCC*mec*IVb	493	
Type IVc-F Type IVc-R	-ACAATATTTGTATTATCGGAGAGC- -TTGGTATGAGGTATTGCTGG-	SCC*mec*IVc	200	
Type IVd-F Type IVd-R	-CTCAAAATACGGACCCCAATACA- -TGCTCCAGTAATTGCTAAAG-	SCC*mec*IVd	881	
Type V-F TypeV-R	-GAACATTGTTACTTAAATGAGCG- -TGAAAGTTGTACCCTTGACACC-	SCC*mec* V	325	
MecA147-F MecA147-R	-GTGAAGATATACCAAGTGATT- -ATGCGCTATAGATTGAAAGGAT-	*mec*A	147	
***Spa* typing**				
SPA 1	-GATTTTAGTATTGCAATACATAATTCG-	114-140		Schmitz *et al*., 1998
SPA 2	-CCACCAAATACAGTTGTACCG-	1702-1682		
SPA 3	-CTTTGGATGAAGCCGTTGCGTTG-	1088-1066		
***Spa* sequencing**				
1095F	-AGACGATCCTTCGGTGAGC-	NA	NA	Larsen *et al*., 2008
1517R	-GCTTTTGCAATGTCATTTACTG-	NA	NA	
***Agr* typing**				
Pan-*agr*B (F)	-ATGCACATGGTGCACATGC-	*agr*B	**-**	Shopsin *et al*., 2003
*agr* I (R)	-GTCACAAGTACTATAAGCTGCGAT-	*agr*D	440	
*agr* II (R)	-GTATTACTAATTGAAAAGTGCCATAGC-	*agr*C	572	
*agr* III (R)	-CTGTTGAAAAAGTCAACTAAAAGCTC-	*agr*D	406	
*agr* IV (R)	-CGATAATGCCGTAATACCCG-	*agr*C	588	
**Multilocus sequence typing**				
*arcC*-fd *arcC*-Rv	-TTGATTCACCAGCGCGTATTGTC- -AGGTATCTGCTTCAATCAGCG-	Carbamate kinase (*arc*C)	500	Enright *et al*., 2000
*aroE*-fd *aroE*-Rv	-ATCGGAAATCCTATTTCACATTC- -GGTGTTGTATTAATAACGATATC-	Shikimate dehydrogenase (*aro*E)	500	
*glpF*-fd *glpF*-Rv	-TGGTAAAATCGCATGTCCAATTC- -CTAGGAACTGCAATCTTAATCC-	Glycerol kinase (*glp*F)	500	
*gmk*-fd *gmk*-Rv	-ATCGTTTTATCGGGACCATC- -TCATTAACTACAACGTAATCGTA-	Guanylate kinase (*gmk*)	500	
*pta*-fd *pta*-Rv	-GTTAAAATCGTATTACCTGAAGG- -GACCCTTTTGTTGAAAAGCTTAA-	Phosphate acetyltransferase (*pta*)	500	
*tpi*-fd *tpi*-Rv	-TCGTTCATTCTGAACGTCGTGAA- -TTTGCACCTTCTAACAATTGTAC-	Triosephosphate isomerase (*tpi*)	500	
*yqiL*-fd *yqiL*-Rv	-CAGCATACAGGACACCTATTGGC- -CGTTGAGGAATCGATACTGGAAC-	Acetyl coenzyme A acetyltransferase (*yqi*L)	500	

NA: Not available

**Table 2:** Frequency of MRSA isolation from clinical specimen and the distribution of the *agr* groups, the *spa* clusters, SCC*mec* types and Pulse field gel electrophoresis clusters among the isolates.

***spa* typing**																														
**Clusters**	**A** 23.5% (44/187)		**B** 22.9% (43/187)		**C** 3.7% (7/187)		**D** 10.2% (19/187)		**E** 13.4% (25/187)		**F** 1.6% (3/187)	**G** 8.6% (16/187)			**H** 2.1% (4/187)		**I** 1.6% (3/187)		**J** 3.2% (6/187)		**K** 4.3% (8/187)				**L** 2.7% (5/187)	Outliers 2.1% (4/187)			Untypeable 2.6% (5/187)	
*agr* typing																														
***agr* types**	*agr*I 84.4% (163/193)						*agr*II 4.7% (9/193)							*agr*III 7.3% (14/193)									*agr*I and III 3.6% (7/193)							
**SCC*mec* typing**																														
**SCC*mec* types**		SCC*mec* type I 1.1% (1/92)		SCC*mec* type II 65.2% (60/92)				SCC*mercury* 33A 7% (31/92)		SCC*mec*type IVa 4% (2/50)			SCC*mec*type IVb 2% (1/50)				SCC*mec*type IVd 94% (47/50)					SCC*mec* type II+IVc 4.7% (9/193)						Not typeable 22.3% (43/193)		
**Classification**		HA-MRSA 47.7% (92/193)								CA-MRSA 25.9% (50/193)												Unknown						Unknown		
**Pulsed field gel electrophoresis typing**																														
**Pulsotypes**			A 57.6% (110/191)			A1-A6 29.3% (56/191)	B 4% (8/191)		C 0.5% (1/191)		D 0.5% (1/191)		E 0.5% (1/191)			F 0.5% (1/191)		G 0.5% (1/191)		H 0.5% (1/191)				I 2% (4/191)			J 5% (9/191)			K 0.5% (1/191)

NB: The table is not meant to show correlations between the typing methods.

**Table 3:** The genetic profile of the ten representative isolates for MLST typing and the Ridom sequence *spa* types including their geographic distribution

Isolate number	PVL		SCC*mec*type	*spa* Cluster	*spa* sequence types		*agr* Group		PFGE	**MLST typing**			**Geographic distribution** **(http://spaserver.ridom.de)**
										**ST**	**Allelic** **Profile**	**CC**	
2	Neg		IVd	D	NA		I		E	NA	3-3-1-1-4-88-?	**??**	NA
71	Pos		V	H	891		I		A3	22	7-6-1-5-8-8-6	22	Europe, Indonesia, SA and Canada
100	Neg		IVd	F	1257		I		A6	612	3-3-1-1-4-88-83	8	SA and Austraila
131	Neg		II+SCC*mecury*	A	037		III		A	239	2-3-1-1-4-4-3	8	Asia, Austraila, SA, South America and Europe
133	Neg		Iva	A	037		I		A	239	2-3-1-1-4-4-3	8	Asia, Austraila, SA, South America and Europe
134	Neg		IVd	NT	NA		I		B2	NT	?-?-1-1-?-4-83-	NT	NA
143	Neg		Iva	D	1257		III		B	612	3-3-1-1-4-88-83	8	SA and Austraila
165	Neg		NT	NT	NA		I		I	NT	?-3-1-1-?-4-3	NT	NA
183	Neg		I	S	012		I, III		J	36	2-2-2-2-3-3-2	30	USA, UK, Austraila, Canada and SA
**Ridom sequence *spa* types, sequence types and geographical distribution of *spa* types identified in this study**													
***Spa* types**		**Ridom sequence of *spa* types**				**Sequence type**					**Geographic distribution** **(http://spaserver.ridom.de)**		
t012		15-12-16-02-16-02-25-17-24-24				ST36		Australia, Belgium, Canada, Cyprus, Denmark, Finland, France, Germany, Iceland, Italy, Jordan, Latvia, Lebanon, Netherlands, New York, Norway, Poland, SA, Spain, Sweden, Switzerland, UK and USA					
t037		15-12-16-02-25-17-24				ST239		Australia, Belgium, Bulgaria, Canada, China, Croatia, Denmark, France, Germany, Iceland, Italy, Jordan, Latvia, Lebanon, Malaysia, Netherlands, New York, Norway, Poland, South Africa, Spain, Sweden, Switzerland, Taiwan and UK					
t891		26-23-13-23-31-05-17-25-17-25-28				ST22		Denmark, Finland, Germany, Norway, South Africa, Sweden and Switzerland					
t1257		11-19-34-05-17-34-24-34-22-25				ST612		Denmark, Germany, Norway and South Africa					

NT: Not typeable because numbers replaced by question mark (?) could not be found on the MLST database; NA: Not applicable; CC: clonal complexes; ST: Sequence types; PVL: Panton Valentine Leukocidin

**Table 4:** Vitek2 automated system (bioMérieux, Mary l'Etoile, France) antimicrobial susceptibility testing (AST) for the ten representative isolates for spa sequencing and MLST typing.

Number	ISOLATE IDENTITY/GENOTYPE	VITEK2 AUTOMATED SYSTEM (SYSTEM (bioMérieux, Mary l'Etoile, France) RESULT	RESISTANT (R) ANTIBIOTICS (µg/ml)	SUSCEPTIBLE (S) ANTIBIOTICS (µg/ml)	INTERMIDIATE (I) ANTIBIOTICS (µg/ml)
1	2Spa cluster A-SCC*mec* I-*agr* I-PFGE A3	MRSA	Clindamycin (≥ 8), Erythromycin (≥ 8), Fusidic Acid (≥ 32), Oxacillin (≥ 4), Rifampicin (≥ 32), Teicoplanin (≥ 32), Tetracycline (8) and Vancomycin (≥ 32)	Ciprofloxacin (≥ 0.5), Gentamicin (≥0.5), Moxifloxacin (≥ 0.25), Tigecycline (0.5) and Trimethoprim-Sulfamethoxazole (≥10)	
2	71 Spa cluster D-SCCmec IV-*agr* I-PFGE A3	MRSA	Clindamycin (≥ 8), Erythromycin (≥ 8), Fusidic Acid (≥ 32), Gentamicin (≥ 16), Rifampicin (≥ 32), Teicoplanin (≥ 32), Tetracycline (≥ 16), Trimethoprim-Sulfamethoxazole (≥ 320), Oxacillin (≥ 4) and Vancomycin (≥ 32)	Ciprofloxacin (≥ 0.5), Moxifloxacin (≥ 0.25) and Tigecycline (0.5)	
3	100 Spa cluster A-SCC*mec* IVd-*agr* I-PFGE A3	MRSA	Ciprofloxacin (≥ 8), Clindamycin (≥ 0.25), Erythromycin (≥ 8), Gentamicin (≥ 16), Tetracycline (≥ 16), Trimethoprim-Sulfamethoxazole (≥ 320), Oxacillin (≥ 4) and Rifampicin (≥ 32)	Fusidic Acid (≥ 0.5), Linezolid (1), Moxifloxacin (1), Mupirocin (≥ 2), Teicoplanin (≥ 0.5), Tigecycline (≥ 0.12) and Vancomycin (1)	
4	131 SCC*mec* IVd-*agr* I-PFGE A	MRSA	Ciprofloxacin (≥ 8), Clindamycin (≥ 0.25), Erythromycin (≥ 8), Gentamicin (≥ 16), Moxifloxacin (2), Oxacillin (≥ 4), Tetracycline (≥ 16) and Trimethoprim-Sulfamethoxazole (≥ 320)	Fusidic Acid (≥ 0.25), Linezolid (2), Rifampicin (≥ 0.5), Teicoplanin (≥ 0.5), Tigecycline (0.25) and Vancomycin (2)	
5	133 SCC*mec* IVa-*agr* I-PFGE A	MRSA	Ciprofloxacin (≥ 8), Clindamycin (≥ 0.25), Erythromycin (≥ 8), Gentamicin (≥ 16), Oxacillin (≥ 4), Tetracycline (≥ 16) and Trimethoprim-Sulfamethoxazole (≥ 320)	Fusidic Acid (≥ 0.5), Linezolid (2), Moxifloxacin (1), Rifampicin (≥ 0.5), Teicoplanin (≥ 0.5), Tigecycline (0.25) and Vancomycin (≥ 0.5)	
6	134 SCC*mec* IVd-*agr* I-PFGE B2	MRSA	Clindamycin (≥ 0.25), Erythromycin (≥ 8) , Gentamicin (≥ 16), Oxacillin (≥ 4), Tetracycline (≥ 4) and Trimethoprim-Sulfamethoxazole (80)	Ciprofloxacin (≥ 0.5), Fusidic Acid (≥ 0.5), Linezolid (1), Moxifloxacin (≥ 0.25), Mupirocin (≥2), Teicoplanin (2), Tigecycline (0.25) and Vancomycin (2)	
7	143 Spa cluster A-SCC*mec* IVa-*agr*-III-PFGE B	MRSA	Ciprofloxacin (≥ 8), Clindamycin (≥ 0.25), Erythromycin (≥ 8), Gentamicin (≥ 16), Oxacillin(≥ 4) , Rifampicin (≥ 32), Tetracycline (≥ 16) and Trimethoprim-Sulfamethoxazole (≥ 320)	Fusidic Acid (≥ 0.5), Linezolid (2), Moxifloxacin (≥ 2) , Mupirocin (≥ 2), Teicoplanin (≥ 0.5), Tigecycline (≥ 0.12) and Vancomycin (≥ 0.5)	
8	165 Not typed	MRSA	Clindamycin (≥ 0.25), Erythromycin (≥ 8), Gentamicin (≥ 16), Oxacillin (≥ 4), Rifampicin (≥ 32), Tetracycline (2) and Trimethoprim-Sulfamethoxazole (160)	Linezolid (1), Moxifloxacin (0.5),Teicoplanin (8), Tigecycline (≥ 0.12) and Vancomycin (2)	Ciprofloxacin (2) and Fusidic Acid (4)
9	183 Spa cluster I-SCC*mec* II-*agr* I, III-PFGE J	MRSA	Ciprofloxacin (≥ 8), Clindamycin (≥ 8), Erythromycin (≥ 8) , Moxifloxacin (≥ 8) and Oxacillin (≥ 8)	Fusidic Acid (≥ 0.25) , Gentamicin (≥ 0.5), Linezolid (≥ 8), Tetracycline (≥ 1), Teicoplanin (2), Tigecycline (≥ 0.5), Rifampicin (≥ 0.5) and Vancomycin (≥ 0.5)	

**Table 5:** MRSA genotypes with the highest recorded frequency of occurrence.

*GenoCluster	**Frequency**
Spa Cluster A-*SCCmercury*-*agr* I	14.5%; (28/193)
Spa Cluster B-SCC*mec* I-*agr* I	19.2%; (37/193)
Spa Cluster C-SCC*mercury*-*agr* I	2.1%; (4/193)
Spa Cluster D-SCC*mec* IV-*agr* I	6.7%; (13/193)
Spa Cluster E-SCC*mercury*-*agr* I	7.8%; (15/193)
Spa Cluster F-SCC*mec* IV-*agr* I	0.5%; (1/193)
Spa Cluster F-SCC*mec* II + SCC*mercury*-*agr* I	0.5%; (1/193)
Spa Cluster F-SCC*mercury*-*agr* I	0.5%; (1/193)
Spa Cluster G-SCC*mercury*-*agr* I	4.2%; (8/193)
Spa Cluster H-SCC*mercury*-*agr* I	1%; (2/193)
Spa Cluster H-SCC*mec* II + SCC*mercury*-*agr* I	1%; (2/193)
Spa Cluster I-SCC*mec* II-*agr* I, III	1%; (2/193)
Spa Cluster J-SCC*mec* II + SCC*mec* IV-*agr* III	2.1%; (4/193)
Spa Cluster K-SCC*mec* II + SCC*mercury*-*agr* I	1%; (2/193)
Spa Cluster L-SCC*mercury*-*agr* I	1.6%; (3/193)

*Genotype with the highest frequency of occurrence. Details of other genotype can be found in Table (**S1**) NB: The pulsotype (PFGE) classification was not included as no significant diversity was observed within this category
