# Supplementary material for: Trends in the Genetic Background of Methicillin-Resistant Staphylococcus Aureus Clinical Isolates in a South African Hospital: An Institutional-Based Observational Study
Source: Open Microbiol J. 2017 Nov 30;11:339–51. doi: 10.2174/1874285801711010339 (PMC5737024; doi:10.2174/1874285801711010339)
Supplement: Supplementary file 1 [file TOMICROJ-11-339_SD1.pdf]

Send Orders for Reprints to reprints@benthamscience.ae

BENTHAM OPEN

CrossMark

The Open Microbiology Journal

Supplementary Material

Content list available at: www.benthamopen.com/TOMICROJ/

DOI: 10.2174/1874285801711010339

Trends in the Genetic Background of Methicillin-Resistant *Staphylococcus Aureus* Clinical Isolates in a South African Hospital: An Institutional-Based Observational Study

John F. Antiabong, Marleen M. Kock, Tsidiso G. Maphanga, Adeola M. Salawu, Nontombi M. Mbelle and Marthie M. Ehlers*

Department of Medical Microbiology, University of Pretoria, Gauteng, South Africa

SUPPORTING INFORMATION

Table (**S1**) Combined patient information and molecular typing data of the 193 MRSA isolates obtained from the Steve Biko Academic Hospital. Summarily, the distribution of MRSA strains among the different clinical conditions was rarely dependent on the genetic backbone or genotype. The corresponding examples are presented as follows (Details of the table can be found below the description:

1. A particular genotype type could be associated with multiple hospital units and could be of CA-MRSA and /or HA-MRSA genotype: PFGE Pulsotype-J was detected in Surgery and Trauma, Paediatric urology, High Care Medical, and Orthopaedic units.
2. Different PFGE pulsotypes could be associated with a treatment material, such as the central venous pressure catheter (CVP) tip in different hospital units and could be of CA-MRSA and /or HA-MRSA genotype:pulsotypes-A1, A3, A4, A6, and F were associated with the CVP tip used in General paediatric, Neurology, Paediatric Surgery, High Care Medical, Paediatric and Surgery and Trauma unit.
3. Isolates of different PFGE pulsotypes were detected in multiple hospital units and could be of CA-MRSA and /or HA-MRSA genotype: isolates of PFGE pulsotypes-A1, A3, A4, A5, B1, I, and J were detected in the Orthopaedic unit.
4. A PFGE pulsotype could be either CA-MRSA or HA-MRSA genotype. This observation was not limited to any specific PFGE pulsotype: CA-MRSA and HA-MRSA genotypes were detected among pulsotypes-A1, A3, A4, A6 and J.
5. An agr type could be either CA-MRSA or HA-MRSA genotype. This observation was not limited to any specific PFGE pulsotype: CA-MRSA and HA-MRSA genotypes were detected among isolates with agr types I to III.

Table S1. Combined patient information and molecular typing data of the 193 MRSA isolates obtained from the steve biko academic hospital.

Isolates ID	Gender	Age	Clinical wards	Specimen type	*spa* cluster	SCC*mec* type	*agr* group	PFGE pulsotypes	HA-MRSA or CA-MRSA
35	M	1y2m	PS	CVP tip	E	I	II	A3	HA-MRSA
52	M	22 y	Neurosurgical	CVP tip	NT	I	I	F	HA-MRSA
54	M	1y2m	PS	CVP tip	K	I	II	A3	HA-MRSA
161	M	60 y	MP	Luki	B	I	II	A3	HA-MRSA

1874-2858/17 2017 Bentham Open

Table S1 contd.....

Isolates ID	Gender	Age	Clinical wards	Specimen type	*spa* cluster	SCC*mec* type	*agr* group	PFGE pulsotypes	HA-MRSA or CA-MRSA
4	M	28 y	Neurosurgical	BC	NT	II	I	NT	HA-MRSA
32	M	45 m	HCM	BC	B	II	III	A5	HA-MRSA
88	M	49 y	ICU	BC	E	II	I, III	A	HA-MRSA
106	M	7 m	Paediatric ICU	BC	G	II	I	A	HA-MRSA
166	M	8 y	Paediatrics	Pus swab	B	II	I	J	HA-MRSA
170	M	16 y	OP	Pus swab	B	II	I	NT	HA-MRSA
171	M	16 y	OP	Pus swab	B	II	I	I	HA-MRSA
183	M	16 y	OP	Pus swab	I	II	I, III	J	HA-MRSA
57	M	52 y	OP	Tissue	K	II	III	A4	HA-MRSA
84	M	39 y	OP	Tissue	J	II	III	A1	HA-MRSA
45	M	1 d	Baby room ICU	BC	L	II+SCCmercury	II	A	HA-MRSA
51	M	56 y	Cardiothoracic	BC	K	II+SCCmercury	I	A	HA-MRSA
53	M	38 y	ST	BC	K	II+SCCmercury	I	A	HA-MRSA
119	M	52 y	Surgery	BC	G	II+SCCmercury	I	A	HA-MRSA
126	M	6 m	Paediatric ICU	BC	B	II+SCCmercury	II	A6	HA-MRSA
86	M	67 y	NP	CVP tip	E	II+SCCmercury	I, III	A1	HA-MRSA
130	M	49 y	ST	Luki	B	II+SCCmercury	I	A4	CA-MRSA
72	M	48 y	CS	Pus swab	B	II+SCCmercury	I	A	HA-MRSA
94	M	47 y	OP	Pus swab	A	II+SCCmercury	I	A	HA-MRSA
115	M	31 y	ARV	Pus swab	G	II+SCCmercury	I	A	HA-MRSA
135	M	28 y	HCM	Pus swab	B	II+SCCmercury	I	A	HA-MRSA
65	M	20 y	Neurological	Urine	F	II+SCCmercury	I	A	HA-MRSA
122	M	23 d	Paediatric ICU	BC	B	II+SCCmercuy	I	A4	HA-MRSA
144	M	40 d	Neonatal ICU	BC	A	II+SCCmercy	I	A	HA-MRSA
155	M	7 d	Paediatric ICU	BC	B	II+SCCmercy	I	A	HA-MRSA
153	M	52 y	ST	CVP tip	B	II+SCCmerry	I	A	HA-MRSA
158	M	57 y	HCM	CVP tip	B	II+SCCmerry	I	A	HA-MRSA
172	M	19 d	PS	CVP tip	Outlier	II+SCCmerry	I	A6	HA-MRSA
156	M	49 y	ST	Luki	B	II+SCCmerry	I	A	HA-MRSA
159	M	60y	MP	Luki	B	II+SCCmerry	I	A	HA-MRSA
152	M	2y2m	ARV	Pus swab	B	II+SCCmerry	I	A	HA-MRSA
149	M	39 y	OP	Tissue	B	II+SCCmerry	I	A	HA-MRSA
179	M	16 y	OP	Pus swab	D	IV	I	A	CA-MRSA
175	M	16 y	OP	Tissue	B	IV	I	B1	CA-MRSA
133	M	27 y	HCM	BC	B	IVa	I	A	CA-MRSA
143	M	45 y	ST	Pus swab	A	IVa	III	B	CA-MRSA
2	M	35 y	Urology	BC	A	IVd	I	E	CA-MRSA
39	M	92 d	Coronary ICU	BC	D	IVd	I	A4	CA-MRSA
47	M	15 y	Nephrology	BC	L	IVd	I	B	CA-MRSA
55	M	13 d	Neonatal ICU	BC	Outlier	IVd	I	A5	CA-MRSA
58	M	16 d	Paediatric ICU	BC	K	IVd	I	A1	CA-MRSA
61	M	27 y	ST	BC	J	IVd	III	A4	CA-MRSA
100	M	52 y	Nephrology	BC	A	IVd	I	A6	CA-MRSA
132	M	60 y	IM	BC	B	IVd	I	H	CA-MRSA
142	M	27 y	Surgery male	Catheter tip	D	IVd	I, III	A3	CA-MRSA
31	M	49 y	Neurosurgery	CVP tip	A	IVd	III	D	CA-MRSA
38	M	26 y	HCM	CVP tip	D	IVd	I	A4	CA-MRSA
41	M	10 m	GP	CVP tip	D	IVd	I	A6	CA-MRSA
63	M	57 y	HCM	CVP tip	F	IVd	I	A	CA-MRSA
137	M	70 y	IM	CVP tip	A	IVd	I	B1	CA-MRSA
49	M	50 y	Neurosurgical	Luki	L	IVd	I	A5	CA-MRSA
98	M	40 y	CS	Luki	A	IVd	I	A4	CA-MRSA
138	M	35 y	Main casualty	Luki	NT	IVd	I	I	CA-MRSA

Table S1 contd.....

Isolates ID	Gender	Age	Clinical wards	Specimen type	*spa* cluster	SCC*mec* type	*agr* group	PFGE pulsotypes	HA-MRSA or CA-MRSA
24	M	35 y	OP	Pus swab	E	IVd	I	A3	CA-MRSA
26	M	33 y	OP	Pus swab	E	IVd	I	A	CA-MRSA
73	M	16 y	OP	Pus swab	E	IVd	I	A	CA-MRSA
90	M	31 y	NP	Pus swab	G	IVd	I	A6	CA-MRSA
101	M	28 y	HCM	Pus swab	B	IVd	I	B	CA-MRSA
117	M	49 y	Nephrology	Pus swab	G	IVd	I	A	CA-MRSA
146	M	38 y	OP	Pus swab	B	IVd	I	A	CA-MRSA
42	M	2 y	Paediatrics	Sputum	D	IVd	I	A4	CA-MRSA
76	M	27 y	MP	Sputum	E	IVd	I	A4	HA-MRSA
19	M	65 y	OP	Tissue	A	IVd	I	A5	CA-MRSA
194	M	31 y	Main casualty	Sputum	Outlier	NT	I	A	NT
164	M	1y2m	PS	CSF	B	NT	II	A3	NT
163	M	38 y	OP	Tissue	A	NT	I	A	NT
3	M	28 y	Neurosurgical	BC	A	SCCmercury	I	A	HA-MRSA
5	M	28 y	ST	BC	A	SCCmercury	I	A	HA-MRSA
15	M	4 y	Ward 60	BC	A	SCCmercury	I	A	HA-MRSA
30	M	2y6m	PS	BC	E	SCCmercury	I	A	HA-MRSA
43	M	45 y	Short stay	BC	L	SCCmercury	I	A	HA-MRSA
44	M	9 d	Neonatal ICU	BC	K	SCCmercury	I	A	HA-MRSA
48	M	44 y	ST	BC	L	SCCmercury	I	A	HA-MRSA
50	M	25 d	Paediatric ICU	BC	K	SCCmercury	I	A	HA-MRSA
59	M	25 y	ST	BC	D	SCCmercury	I	A2	HA-MRSA
60	M	25 y	ST	BC	H	SCCmercury	I	A	HA-MRSA
67	M	4 d	Paediatric ICU	BC	C	SCCmercury	I	A	HA-MRSA
75	M	49 y	ICU	BC	E	SCCmercury	I	K	HA-MRSA
77	M	49 y	ICU	BC	E	SCCmercury	I	A	HA-MRSA
92	M	7 d	Paediatric ICU	BC	E	SCCmercury	I	A	HA-MRSA
96	M	29 y	Surgery burns	BC	A	SCCmercury	I	A	HA-MRSA
110	M	13 y	Neurosurgical	BC	G	SCCmercury	I	A3	HA-MRSA
114	M	4 d	Paediatric ICU	BC	G	SCCmercury	I	A	HA-MRSA
129	M	35 y	HCM	BC	B	SCCmercury	I	A	HA-MRSA
169	M	32 y	HCM	BC	B	SCCmercury	I	A	HA-MRSA
40	M	27 y	ST	CVP tip	C	SCCmercury	I	A	CA-MRSA
17	M	63 d	Paediatric ICU	CVP tip	A	SCCmercury	I	A1	HA-MRSA
18	M	2 y	Neurosurgery	CVP tip	A	SCCmercury	I	A1	HA-MRSA
78	M	52 y	HCM	CVP tip	E	SCCmercury	I	A	HA-MRSA
136	M	31 y	Neurosurgical	CVP tip	B	SCCmercury	I	A1	HA-MRSA
16	M	2y6m	Neurosurgical	Luki	A	SCCmercury	I	A1	HA-MRSA
68	M	60 y	MP	Luki	C	SCCmercury	I	A	HA-MRSA
69	M	60 y	MP	Luki	C	SCCmercury	I	A	HA-MRSA
120	M	78 y	CS	Luki	G	SCCmercury	I	A	HA-MRSA
7	M	29 y	IM	Pus swab	A	SCCmercury	I	A	HA-MRSA
46	M	17 d	Neonatal ICU	Pus swab	Outlier	SCCmercury	I	B	HA-MRSA
74	M	29 y	OP	Pus swab	E	SCCmercury	I	A3	HA-MRSA
83	M	48 y	Urology	Pus swab	E	SCCmercury	I	A5	HA-MRSA
99	M	8 y	Paediatrics	Pus swab	A	SCCmercury	I	A	HA-MRSA
118	M	51 y	Main casualty	Pus swab	G	SCCmercury	I	A	HA-MRSA
147	M	2y2m	ARV	Pus swab	B	SCCmercury	I	A	HA-MRSA
34	M	22 y	OP	Tissue	A	SCCmercury	I	A	HA-MRSA
37	M	22 y	OP	Tissue	B	SCCmercury	I	A	HA-MRSA
79	M	10 m	GP	Tissue	E	SCCmercury	I	A	HA-MRSA
10	M	29 y	IM	Urine	A	SCCmercury	I	A	HA-MRSA
64	M	50 y	Urology	Urine	F	SCCmercury	I	A	HA-MRSA

Table S1 contd.....

Isolates ID	Gender	Age	Clinical wards	Specimen type	*spa* cluster	SCC*mec* type	*agr* group	PFGE pulsotypes	HA-MRSA or CA-MRSA
1	F	27 d	PS	Pus swab	A	I	II	A3	HA-MRSA
141	F	46 y	ST	BC	K	II	I	J	HA-MRSA
184	F	47 y	ST	Pus swab	I	II	I,III	J	HA-MRSA
185	F	29 y	Main casualty	Tissue	A	II	I	A	HA-MRSA
80	F	81 y	ST	Urine	J	II	III	A5	HA-MRSA
190	F	47 y	ST	BC	J	II+IVc	III	J	CA-MRSA
191	F	49 y	HCM	BC	J	II+IVc	III	J	CA-MRSA
192	F	47 y	ST	BC	J	II+IVc	III	J	CA-MRSA
193	F	47 y	HCM	BC	J	II+IVc	I,III	J	CA-MRSA
91	F	22 d	Neonatal ICU	BC	H	II+SCCmercury	I	A	HA-MRSA
103	F	6 d	Neonatal ICU	BC	D	II+SCCmercury	I	A	HA-MRSA
112	F	77 y	HCM	BC	G	II+SCCmercury	I	A	HA-MRSA
140	F	40 d	Neonatal ICU	Briviac tip	H	II+SCCmercury	I	A	HA-MRSA
56	F	73 y	IM	CVP tip	C	II+SCCmercury	III	A	HA-MRSA
109	F	16 y	NP	NP	G	II+SCCmercury	I	A	HA-MRSA
131	F	42 y	ST	NP	B	II+SCCmercury	II	A	HA-MRSA
66	F	14 y	Oncology	Pus swab	D	II+SCCmercury	I	A	HA-MRSA
70	F	69 y	OP	Pus swab	C	II+SCCmercury	I	A	HA-MRSA
81	F	81 y	OP	Pus swab	D	II+SCCmercury	I	A	HA-MRSA
82	F	81 y	OP	Pus swab	E	II+SCCmercury	I	A	HA-MRSA
128	F	65 y	OP	Pus swab	B	II+SCCmercury	I	A	HA-MRSA
148	F	9 d	Paediatric	BC	B	II+SCCmerry	I	A	HA-MRSA
154	F	26 d	Neonatal ICU	BC	B	II+SCCmerry	I	A	HA-MRSA
145	F	28 y	Gynaecology	Pus swab	B	II+SCCmerry	I	A3	HA-MRSA
151	F	60 y	HCM	Pus swab	B	II+SCCmerry	I	A	HA-MRSA
150	F	64 y	IM	Sputum	B	II+SCCmerry	I	A	HA-MRSA
160	F	24y	Main casualty	Tissue	B	II+SCCmerry	I	A	HA-MRSA
157	F	46 y	ST	Urine	B	II+SCCmerry	I	A	HA-MRSA
173	F	1 y	Paediatrics	BC	A	IV	I	A	CA-MRSA
176	F	72 y	HCM	Sputum	D	IV	I	A5	CA-MRSA
93	F	37 y	Neurology	BC	D	IVd	I	A5	CA-MRSA
95	F	62 y	ST	BC	A	IVd	I	A1	CA-MRSA
104	F	61 y	OP	BC	D	IVd	I	B	CA-MRSA
105	F	5 m	Neonatal ICU	BC	D	IVd	I	A6	CA-MRSA
189	F	38 y	Neurology	BC	D	IVd	I	A	CA-MRSA
123	F	4 y	Neonatal ICU	Briviac tip	A	IVd	I	A3	CA-MRSA
108	F	24 y	Neurosurgical	CVP tip	G	IVd	I	A6	CA-MRSA
139	F	52 d	PS	CVP tip	NT	IVd	I	I	CA-MRSA
13	F	58 y	MP	Luki	D	IVd	I	A5	CA-MRSA
62	F	14 y	Oncology	Pus swab	E	IVd	I	A	CA-MRSA
85	F	14 y	Oncology	Pus swab	Outlier	IVd	I	G	CA-MRSA
134	F	78 y	UG	Pus swab	NT	IVd	I	B2	CA-MRSA
124	F	4y3m	PP	Sputum	B	IVd	I	A5	CA-MRSA
29	F	23 y	Nephrology	Urine	E	IVd	I	C	CA-MRSA
165	F	64 y	MP	Sputum	NT	NT	I	I	NT
6	F	38 y	ST	BC	A	SCCmercury	I	A	HA-MRSA
8	F	38 y	ST	BC	A	SCCmercury	I	A	HA-MRSA
9	F	38 y	ST	BC	A	SCCmercury	I	A	HA-MRSA
11	F	38 y	ST	BC	A	SCCmercury	I	A	HA-MRSA
12	F	38 y	ST	BC	A	SCCmercury	I	A	HA-MRSA
20	F	38 y	ST	BC	E	SCCmercury	I	A	HA-MRSA
22	F	38 y	ST	BC	B	SCCmercury	I	A	HA-MRSA
23	F	7 m	PS	BC	E	SCCmercury	I	A	HA-MRSA

Table S1 contd.....

Isolates ID	Gender	Age	Clinical wards	Specimen type	*spa* cluster	SCC*mec* type	*agr* group	PFGE pulsotypes	HA-MRSA or CA-MRSA
25	F	4y4m	PS	BC	E	SCCmercury	I	A3	HA-MRSA
27	F	47 y	ST	BC	E	SCCmercury	I	A	HA-MRSA
36	F	4 m	ICU	BC	A	SCCmercury	I	A4	HA-MRSA
87	F	35 d	Paediatric ICU	BC	E	SCCmercury	I	A	HA-MRSA
89	F	26 d	Neonatal ICU	BC	E	SCCmercury	I	A	HA-MRSA
121	F	71 y	IM	BC	G	SCCmercury	I	A	HA-MRSA
162	F	75 y	Maxifacial	BC	B	SCCmercury	I	A	HA-MRSA
14	F	38 y	ST	CVP tip	A	SCCmercury	I	A1	HA-MRSA
21	F	38 y	ST	CVP tip	A	SCCmercury	I	A	HA-MRSA
28	F	2y1m	PS	CVP tip	E	SCCmercury	I	A	HA-MRSA
33	F	12 d	ST	CVP tip	A	SCCmercury	I	A	HA-MRSA
178	F	33 y	MP	CVP tip	A	SCCmercury	I	A	HA-MRSA
107	F	42 y	ST	Luki	G	SCCmercury	I	A6	HA-MRSA
113	F	54 y	NP	NP	G	SCCmercury	I	A	CA-MRSA
111	F	1y1m	NP	NP	G	SCCmercury	I	A	HA-MRSA
102	F	39 y	OP	Pus swab	H	SCCmercury	I	A	HA-MRSA
116	F	24 y	GS	Pus swab	B	SCCmercury	I	A	HA-MRSA
127	F	28 d	Neonatal ICU	Pus swab	A	SCCmercury	I	A6	HA-MRSA
167	F	58 y	Neurosurgical	Pus swab	B	SCCmercury	I	A	HA-MRSA
168	F	42 y	Surgery	Pus swab	B	SCCmercury	III	A	HA-MRSA
97	F	39 y	OP	Tissue	D	SCCmercury	I	A	HA-MRSA
177	F	72 y	OP	Tissue	A	SCCmercury	I	A	HA-MRSA
187	F	35 y	Main casualty	Tissue	A	SCCmercury	I	A	HA-MRSA
174	F	20 d	NeonatalICU	BC	B	SCCmercury	II	A4	HA-MRSA
180	F	69 y	OP	Tissue	A	SCCmercury	I, III	A5	HA-MRSA
181	F	30 y	HCM	BC	I	SCCmercury (II+ SCCmercury)	III	J	HA-MRSA
182	F	25 y	Neonatal ICU	BC	A	SCCmercury (II+ SCCmercury)	III	A	HA-MRSA
186	F	29 y	Main casualty	Tissue	A	SCCmercury (II+SCCmercury)	I	A	HA-MRSA
188	F	69 y	OP	Tissue	A	SCCmercury (II+SCCmercury)	I	A	HA-MRSA
71	F	20 y	OP	Pus swab	D	V	I	A3	CA-MRSA

ARV: Anti-retroviral clinic, BC: Blood culture, CVP tip: Central venous pressure tip, CSF: Cerebrospinal fluid, d: Days, F: Female, GS: General surgery, M: Male, ND: Not detected, PS: Paediatric surgery, ICU: Intensive care unit, m: Months, NP: Not provided, ST: Surgery and trauma, NA: Not applicable, IM: Internal medicine, MP: Medical pulmonology, NT: Not typeable, y: Years, Luki: Endotracheal aspirate, OP: Orthopaedic, HCM: High care medical unit, PFGE: Pulsed Field Gel Electrophoresis, *agr*: Staphylococcus agr gene, *spa:* Staphylococcus spa A gene

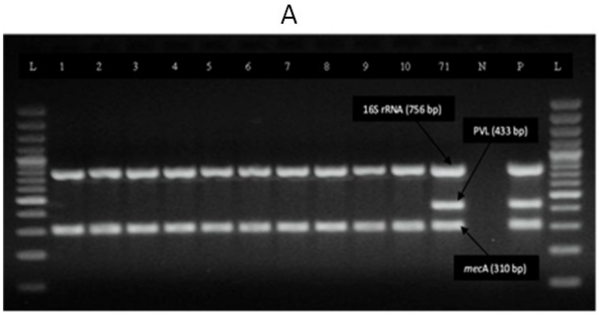

Fig. (S1A). Gel electrophoresis of the M-PCR assay for the detection of the 16S rRNA, mecA and PVL genes of the MRSA isolates. Lanes 1 to 10 show the 16S rRNA (756 bp) and mecA (310 bp). Lane 71 shows the 16S rRNA (756 bp), mecA (310 bp) and PVL (433 bp) genes. N= negative control, P= positive control and L = 100 bp ladder.

B

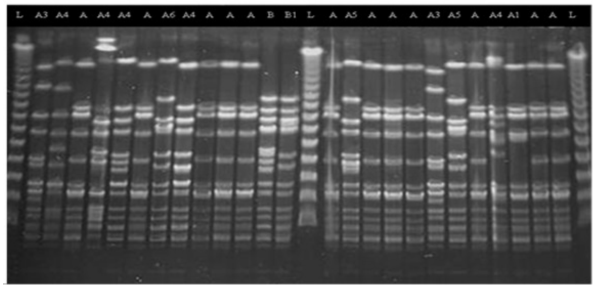

Fig. (S1B). A representative pulsed-field gel electrophoresis fingerprint showing the separation of restriction fragments of the MRSA genome digested with the SmaIenzyme and analysed as described by Tenover *et al.*, (2009), L= 48.5-1,000 kbLambda ladder (Bio-rad, South Africa)

Fig. (**S2**): This is presented as a power point file to allow for detail viewing. Please download online.

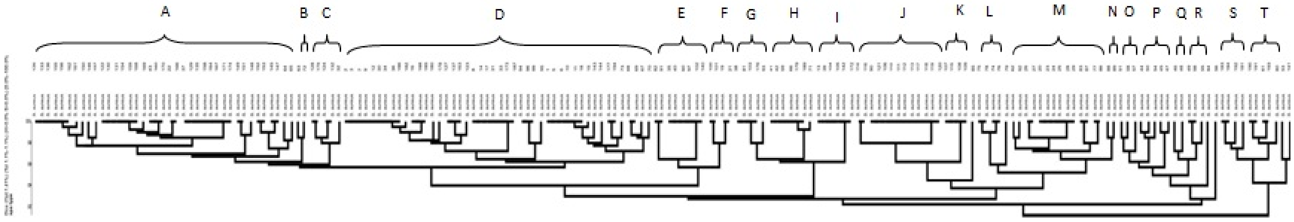

Fig. (S2). UPGMA dendrogramme obtained for spa-clustering analysis, depicting genetic diversity (clusters A to T) of the 187 typeable clinical MRSA isolates obtained from Steve Biko Academic Hospital, Gauteng, South Africa. A 70% similarity cut-off value was used for cluster definition. The following isolates were considered outliers, as they did not fall under a major node within the cut-off value: 35, 40, 44, 50, 53, 60, 79, 82, 84, 85, 91, 102, 140 and 141.

© 2017 Antiabong *et al.*

This is an open access article distributed under the terms of the Creative Commons Attribution 4.0 International Public License (CC-BY 4.0), a copy of which is available at: https://creativecommons.org/licenses/by/4.0/legalcode. This license permits unrestricted use, distribution, and reproduction in any medium, provided the original author and source are credited.
